# Sail training as a blue intervention. Strenghtening hardiness and resilience of adolescents

**DOI:** 10.1192/j.eurpsy.2025.1173

**Published:** 2025-08-26

**Authors:** M. W. Romaniuk, M. Gawrych

**Affiliations:** 1 The Maria Grzegorzewska University, Warsaw, Poland

## Abstract

**Introduction:**

Nature based interventions are becoming more popular in mental health prevention and treatment. Blue therapies are rarely known because of limited access and high costs. The poster presents the results of research on the level of the hardiness and resilience of high sea cruises participants. The study involved 123 people, including 65 girls and 58 boys, and 55 young adults, including 15 women and 39 men.

**Objectives:**

The aim of the study was to assess the impact of blue intervention on the level of the hardiness and resilience of high sea cruises participants.

**Methods:**

Pre-posttest study with questionnaires issued on the first and last day of each cruise. Dispositional Resilience Scale (Bartone et. al., 1989) was used to measure mental hardiness, commitment, openness to challenges and a sense of control of participants. EEA Resilience Scale (Maltby et. al., 2015) was used to measure ecological and engineering resilience and adaptive capacity of partifcipants.

**Results:**

The results show a statistically significant increase in hardiness level. There was also a significant increase in commitment, openness to challenges and a sense of control of participants, which are measured by subscales of the DRS. Significant increase of ecological resilience and adaptive capacity has been noted.

**Conclusions:**

Hardiness and resilience are key protective factors for mental health. Blue interventions can be effetcive ways of mental wellbeing improvement.

**Disclosure of Interest:**

None Declared

